# Are dental professionals ready to deal with medical emergencies in their clinical office? A survey of university hospitals

**DOI:** 10.25122/jml-2022-0012

**Published:** 2022-08

**Authors:** Baraa Issam Abdulrahman, Khaled Mohammed Alasmari, Majed Nasser Alratiq, Fahad Adel Alherab, Mohammed Abdullah Alfantoukh, Abdullah Adel Alherab

**Affiliations:** 1 Department of Oral Maxillofacial Surgery and Diagnostic Sciences, Riyadh Elm University, Riyadh, Saudi Arabia

**Keywords:** medical emergency, emergency drugs, CPR, Saudi Arabia, ER – Emergency, CPR – Cardiopulmonary resuscitation, GP – General practitioner, OMFS – Oral and maxillofacial surgery

## Abstract

This research aimed to assess the availability and need of dental emergency kits in Saudi Arabia university hospitals. A cross-sectional study was conducted among 267 dentists, including undergraduate, dental interns, general dentists, and specialists in 6 university hospitals (private and government colleges). In addition, a closed-ended questionnaire was distributed through emails using the online platform. The data revealed that 49.4% of dentists faced medical emergencies. Out of them, 72.7% said that emergency kits were available in their clinics. Sugar sources and oxygen were most commonly available. On the other hand, 37.8% of dentists handled emergencies independently, 34.5% considered themselves competent with cardiopulmonary resuscitation (CPR), and 28.8% were confident of using emergency (ER) drugs. The most common medical emergencies were vasovagal syncope and hypoglycemia. The emergency kit in dental clinics is relatively available, and the incidence of medical emergencies is relatively minor. However, the competence and confidence of the dentists in tackling an emergency is low, including handling of emergency (ER), knowledge of CPR, and its performance. Therefore, CPR courses should be improved and promoted more widely for this purpose.

## INTRODUCTION

The availability of an emergency kit is necessary in every dental office, and the dental team members must know how to use this kit to handle emergency cases. Although the emergency kit should be easily accessible, dental staff members should periodically check the drugs and materials. A dental team with better knowledge and experience handling the emergency kit will result in better outcomes in an emergency, thus preventing serious situations such as coma and even death [1].

Vasovagal syncope is the most common medical emergency experienced by 53.1% of dentists, followed by hypoglycemia (44.8%), and foreign body aspiration experienced by only 5.5% of dentists. Most participants (74.3%) reported having an emergency kit in their clinics, with more than 70% having oxygen, adrenaline, and glucose readily available. However, one-third of dentists were either not confident or did not know how to use the emergency drugs [2].

Previous research in private clinics in Riyadh showed that 85% of respondents (N=276) have an emergency drug kit in their clinics, but only 55% (N=179) check the emergency drug kit, when considering oral and maxillofacial surgery (OMFS) (N=25, 95%), periodontists (N=12, 86%), pedodontist (N=25, 76%), general practitioner GPs (N=74, 73%), operative dentists (N=29, 51%), endodontists (N=10, 29%), prosthodontists (N=2, 6%), orthodontist (N=1, 5%) and preventive dentists 0% [3].

Another study stated that 92% (N=65) of the offices obtain a thorough medical history preceding the treatment. However, only 11% (N=8) get vital signs for each visit. Using a preparedness percent score (0 to 100), the mean level of preparedness of the office personnel in all surveyed dental offices was 55.2±20. The availability of emergency drugs was 35±35, and equipment 19±22 [4].

Considering this, a study was planned to assess the need for dental emergency kits in various university hospitals. Additionally, the research aimed to evaluate the knowledge of dental professionals on the emergency kit, as well as their preparedness in case a medical emergency occurs in the dental office. Therefore, the specific objective of the study was to check the utilization and awareness related to the dental emergency kit in cases of medical emergency among dental professionals in university hospitals.

## MATERIAL AND METHODS

To fulfill the research objective, the authors planned and executed a cross-sectional study among dentists, including undergraduates, dental interns, general dentists, and specialists in 6 university hospitals (private and government colleges). The study population was composed of all dental professionals from universities. The population was evaluated from February 2020 to February 2021 using a self-developed close-ended questionnaire distributed through email using the SurveyMonkey platform (Annex 1). The questionnaire included the dentists' primary demographic data and attitudes when encountering ER cases.

Descriptive statistics were presented as counts, proportions (%), mean, and standard deviation. The relationship between the emergency cases encountered and the essential demographic characteristics of dentists were assessed using the Pearson Chi-square test/Fisher exact test. A P-value of <0.05 was considered statistically significant. Statistical Software Package SPSS version 22 (IBM Corp. Released 2013) was used for analysis.

## RESULTS

Out of the 267 respondents, a statistically significant majority (65.5%) were below the age of 25 years (X^2^=25.810, df=1, p<0.001). The overall mean age of the sample was 24.9 (±4.8) years.

A statistically significant majority of 57.7% were male dentists (X^2^=6.296, df=1, p=0.012). A statistically significant majority (62.2%) of the respondents were undergraduates (X^2^=213.82, df=3, p<0.001*). Moreover, 42.7% of the respondents were practicing for 2 to 3 years and 31.1% completed more than 3 years of practice (X^2^=11.483, df=2, p=0.003*). Out of the respondents, 51.7% worked in government colleges (X^2^=0.303, df=1, p=0.582) and 89.9% completed or were pursuing their bachelor's degree in Saudi Arabia (X^2^=169.92, df=1, p<0.001*).

**Figure 1:**
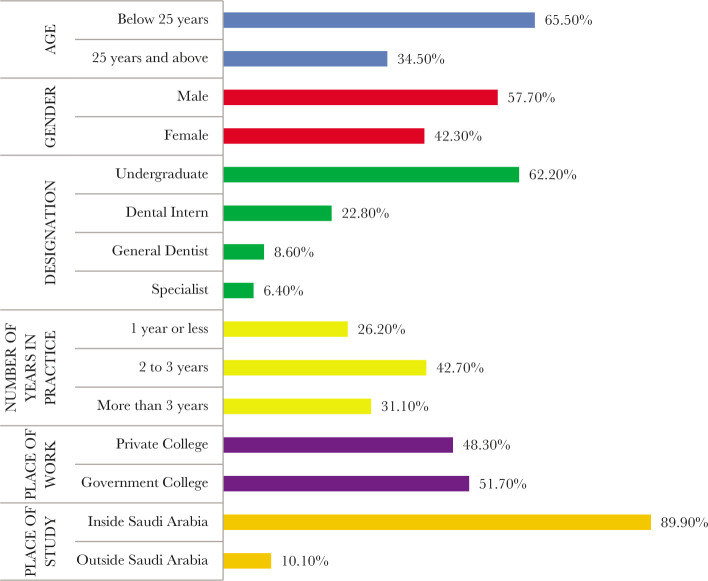
Description of the socio-demographic and professional profile of the dentists (n=267).

Two hundred sixty-seven dentists were recruited for this study. As described in (Figure 1) a majority of them (65.50%) were in the younger age group (<25 years), with the mean age of the sample being 24.9 (SD=4.94). There were more male (57.7%) than female respondents (42.3%), and more than half (62.20%) of the enrolled participants were undergraduate students. The mean practice period in years was 3.07 (SD 2.49), and 42.70% had 2–3 years of experience in practice (Figure 1). Regarding the workplace, approximately 51.7% were in government colleges, while the rest worked at a private set-up (48.30%). Additionally, many dentists got their bachelor's degrees in Saudi Arabia.

135 out of 267 dentists (50.6%) did not face any medical emergency, which is a significant majority (p<0.001) (Table 1). In addition, 194 out of 267 dentists, *i.e*., a statistically significant 72.7% of the dentists, said that ER kits were available in their clinics, while 27.3% reported non-availability of kits (p<0.001).

**Table 1:** Emergency handling experience and competence.

		Frequency	Percent	Chi-Square test P-value
**Availability of ER kit in clinic**	Yes	194	72.7%	p<0.001*
	No	73	27.3%	
**Number of emergencies encountered in the past 3 years**	None	135	50.6%	p<0.001
	1 to 2 cases	85	31.8%	
	3 or more cases	47	17.6%	
**Common emergency drugs available in clinics**	Sugar source	152	59.90%	0.001*
	Oxygen	145	56.60%	0.037*
	Adrenalin	114	42.70%	0.019*
	Aspirin	97	36.30%	p<0.00*
	Nitroglycerine	66	24.70%	p<0.001*
	Diazepam	55	20.60%	p<0.001*
	Salbutamol Inhaler	47	17.60%	p<0.001*
	Diphenhydramine	38	14.20%	p<0.001*
**Handling of emergencies**	Called an ambulance	36	13.50%	p<0.001*
	Called another dentist	70	26.20%	
	Called a physician	60	22.50%	
	Handled it myself	101	37.80%	
**Evaluation of CPR training**	Excellent	33	12.40%	p<0.001*
	Very good	102	38.20%	
	Fair	97	36.30%	
	Poor	18	6.70%	
	Very poor	4	1.50%	
	No training received	13	4.90%	
**Competent with CPR**	Yes	92	34.50%	0.336
	No	78	29.20%	
	Don't know	97	36.30%	
**Confidence about emergency drugs**	Confident	77	28.80%	0.012*
	Not confident	112	41.90%	
	Don't know	78	29.20%	

**Figure 2:**
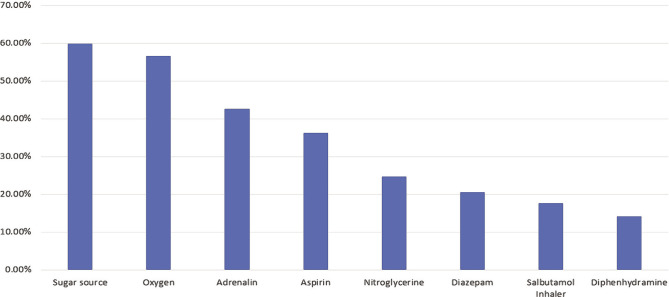
The most common emergency drug available in the clinic.

Glucose (sugar source) was the most commonly available drug reported by a significant number of dentists (N=152, 59.9%), followed by oxygen (N=145, 56.6%). However, other drugs were not commonly available, including adrenaline, for which availability was reported by 114 dentists (42.7%), aspirin (36.3%), nitroglycerine (24.7%), Diazepam (20.6%), salbutamol inhaler (17.6%) and Diphenhydramine (14.2%). Thus, these findings indicate that although most clinics have emergency kits available, drugs apart from sugar sources and oxygen are often not adequately stocked (Table 1) (Figure 2).

Only 101 dentists (37.8%) said that they handled the emergencies on their own, while the remaining 62.2% sought the help of others. Out of these, 60 dentists (22.50%) called for a physician, 70 dentists (26.2%) called for another dentist, and 36 dentists (13.5%) called an ambulance to manage the emergency cases (Table 1).

When considering the evaluation of CPR courses, 50.6% of the dentists reported satisfactory results. Of these, 33 dentists (12.4%) found them excellent, and 102 dentists (38.20%) said the courses were very good. The remaining 49.4% of dentists said the courses were either fair, poor, or very poor. In contrast, 13 dentists (4.9%) said they had not received any CPR training yet.

When considering the competence in performing CPR, 78 dentists (29.2%) did not consider themselves competent, and 97 dentists (36.3%) did not know whether they were competent to perform CPR. Thus, only 92 dentists (34.5%) considered themselves competent with CPR.

Regarding their confidence in administering emergency drugs, 41.9% of the dentists did not consider themselves competent, and 29.2% did not know whether they were competent to perform CPR. On the other hand, 34.5% of the dentists considered themselves competent with CPR. Only 29.2% were confident about using the ER drugs (Table 1).

Upon examination of cross-references, 49 of the 70 dentists (70%) who were in practice for 1 year or less did not encounter any emergency case in the last 3 years, while only 31 of the 83 dentists (37.3%) who were in practice for more than 3 years did not face an emergency in the last 3 years. The association between the number of emergencies encountered and the number of years in practice was statistically significant (p<0.001) (Table 2).

**Table 2:** Association of medical emergencies encountered and years in practice.

		Years in practice			Pearson's Chi-Square test P-value
		1 year or less	2 to 3 years	More than 3 years	
**Number of emergencies encountered**	None	49 (70%)	55 (48.2%)	31 (37.3%)	p<0.001*
	1 to 2 cases	15 (21.4%)	43 (37.7%)	27 (32.5%)	
	3 or more cases	6 (8.6%)	16 (14.0%)	25 (30.1%)	

**Figure 3:**
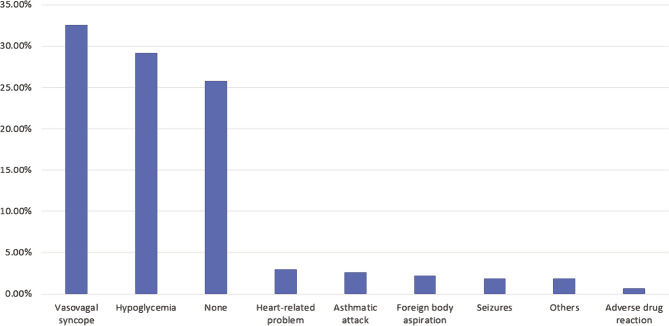
The most common medical emergency in the dental clinic.

The data suggest that vasovagal syncope was the most common medical emergency (N=87, 32.6%), followed by hypoglycemia (N=78, 29.2%). The least common emergency was adverse drug reaction (N=2, 0.7%). The distribution was statistically significant (p<0.001). There was a statistically significant association between common medical emergencies and the number of emergencies faced to date. 54 of the 135 dentists (40%) who did not encounter an emergency to date reported no single common emergency. In comparison, 37 out of 85 dentists (43.5%) who encountered 1–2 cases said vasovagal syncope was the most common emergency, while 19 out of 47 dentists (40.4%) who faced more than three emergencies reported hypoglycemia as the most common emergency (Table 3) (Figure 3).

**Table 3:** Nature of medical emergencies.

		Frequency	Percent	Chi-Square test P-value	Number of ER encountered			Pearson's Chi-Square test P-value
					None	1 to 2 cases	3 or more cases	
**Common medical emergencies**	None	69	25.8%	p<0.001*	54 (40%)	12 (14.1%)	3 (6.4%)	p<0.001*
	Adverse drug reaction	2	0.7%		1 (0.7%)	0 (0%)	1 (2.1%)	
	Asthmatic attack	7	2.6%		2 (1.5%)	2 (2.4%)	3 (6.4%)	
	Foreign body aspiration	6	2.2%		4 (3.0%)	1 (1.2%)	1 (2.1%)	
	Heart-related problem	8	3.0%		5 (3.7%)	2 (2.4%)	1 (2.1%)	
	Hypoglycemia	78	29.2%		29 (21.5%)	30 (35.3%)	19 (40.4%)	
	Seizures	5	1.9%		3 (2.2%)	0 (0%)	2 (4.3%)	
	Vasovagal syncope	87	32.6%		35 (25.9%)	37 (43.5%)	15 (31.9%)	
	Others	5	1.9%		2 (1.5%)	1 (1.2%)	2 (4.3%)	

In clinics where ER kits were not available, 47 out of 73 dentists (64.4%) said they did not encounter an emergency in the past three years, and in clinics with ER kits available, 88 out of 194 dentists (45.4%) did not face an emergency in the past three years, with the association being statistically significant (p=0.002). The low prevalence of emergency cases could be a reason for the lack of equipment in the clinics with ER kits, as highlighted in Table 4.

**Table 4:** Association of emergency kit availability with emergency cases encountered.

			ER kit available		Pearson's Chi-Square test
			Yes	No	
**ER cases encountered in the past 3 years**	None	Count	88	47	0.002*
		%	45.4%	64.4%	
	1 to 2 cases	Count	63	22	
		%	32.5%	30.1%	
	3 or more cases	Count	43	4	
		%	22.2%	5.5%	
**Handling emergencies**	Called an ambulance	Count	21	15	p<0.001*
		%	10.8%	20.5%	
	Called another dentist	Count	38	32	
		%	19.6%	43.8%	
	Called a physician	Count	40	20	
		%	20.6%	27.4%	
	Handled it myself	Count	95	6	
		%	49.0%	8.2%	

In clinics where ER kits were available, 95 out of 194 dentists (49%) said that they handled emergencies on their own without any assistance, whereas in clinics where ER kits were not available. Only 6 out of 73 dentists (8.2%) said that they were able to handle emergencies on their own. The rest required the assistance of either another dentist, a physician or had to call an ambulance. This association was statistically significant (p<0.001), as presented in Table 4.

There was no significant association between age and emergencies encountered (p=0.098). There was a significant association between age and the CPR competence of dentists (p=0.001), where 46 out of 92 dentists (50%) aged 25 years and above said that they had competence in CPR, while only 46 out of 175 dentists (26.3%) aged below 25 years considered themselves competent in CPR. Most dentists aged below 25 considered themselves not competent or unsure about their CPR competence. Age was statistically not associated with confidence in using ER drugs (p=0.055), as seen in Table 5.

**Table 5:** Association of demographic factors with emergency handling experience and competence.

		Emergencies encountered in the past 3 years			Chi Square test P-value	CPR competence			Chi Square test P-value	ER drugs confidence			Chi Square test P-value
		None	1 to 2 cases	3 or more cases		Yes	No	Don't know		Confident	Not confident	Don't know	
**Age**	Below 25 years	50.9%	34.9%	14.3%	0.098	26.3%	32.6%	41.1%	0.001*	24.0%	44.6%	31.4%	0.055 NS*
	25 years and above	44.6%	27.2%	28.3%		50.0%	22.8%	27.2%		38.0%	37.0%	25.0%	
**Gender**	Male	41.6%	37.0%	21.4%	0.024*	40.9%	28.6%	30.5%	0.02*	37.7%	37.7%	24.7%	0.001 S**
	Female	58.4%	25.7%	15.9%		25.7%	30.1%	44.2%		16.8%	47.8%	35.4%	

NS* – Non Significant; S** – Significant.

A significant association was found between gender and the emergencies encountered (p=0.024). Out of the 113 female dentists, 69 (58.4%) did not face an emergency in the past three years, compared to 66 out of 154 male dentists (41.6%). Male dentists encountered more emergency cases than female dentists. A significant association was found between gender and CPR competence (p=0.02), where 63 out of 154 male dentists (40.9%) said that they had competence in CPR, while only 29 out of 113 female dentists (25.7%) considered themselves competent in CPR. Most female dentists (74.3%) considered themselves not competent or unsure about their CPR competence. Gender was statistically associated with confidence in using ER drugs (p=0.001). 58 out of 154 male dentists (37.7%) said they were confident about ER drugs, while only 19 out of 113 female dentists (16.8%) felt confident about ER drugs. The majority of the female dentists (83.2%) considered themselves not confident or unsure about their confidence with ER drugs, as seen in Table 5.

The associations between the evaluation of dentists regarding the CPR training courses and their place of study and work were not significant (p=0.198 and p=0.804, respectively).

Dentists who completed their bachelor's degree in Saudi Arabia expressed a similar self-evaluation of their CPR competence compared to dentists who studied outside the country. The association was not significant (p=0.935). More than 50% of dentists, irrespective of place of study, expressed a lack of competence or doubt regarding their competence with CPR. 57 out of 138 dentists from government colleges (41.3%) expressed more competence in CPR, compared to 35 out of 129 private college dentists (27.1%). A significantly higher number of dentists from private colleges expressed a lack of competence or doubt regarding CPR (p=0.002).

Dentists who completed their bachelor's degree in Saudi Arabia expressed similar self-confidence in using ER drugs as dentists who studied outside the country. The association was not significant (p=0.935). However, more than 50% of dentists, irrespective of place of study, expressed a lack of competence or doubt regarding their confidence with ER drugs. Private college dentists expressed similar self-confidence using ER drugs as government college dentists. The association was not significant (p=0.517). More than 50% of dentists, irrespective of workplace, expressed a lack of competence or doubt regarding their confidence with ER drugs (Table 6).

**Table 6:** Association of the place of study and place of work with emergency competence.

		Place of study		Pearson's Chi-square P-value	Place of work		Pearson's Chi-square P-value
		Inside Saudi Arabia	Outside Saudi Arabia		Private	Government	
**Evaluation CPR training**	Excellent	12.9%	7.4%	0.122, NS	12.4%	12.3%	0.804, NS
	Very good	39.2%	29.6%		34.9%	41.3%	
	Fair	35.4%	44.4%		39.5%	33.3%	
	Poor	7.1%	3.7%		7.8%	5.8%	
	Very poor	1.7%	0%		1.6%	1.4%	
	No training received	3.8%	14.8%		3.9%	5.8%	
**CPR competence**	Yes	34.2%	37.0%	0.935, NS	27.1%	41.3%	0.002, S
	No	29.2%	29.6%		38.8%	20.3%	
	Don't know	36.7%	33.3%		34.1%	38.4%	
**ER drugs confidence**	Confident	27.1%	44.4%	0.112, NS	25.6%	31.9%	0.517, NS
	Not confident	43.8%	25.9%		43.4%	40.6%	
	Don't know	29.2%	29.6%		31.0%	27.5%	

S – Significant; NS – Not Significant.

## DISCUSSION

This study highlighted the preparedness of dentists in case of a medical emergency. A medical emergency was not frequently experienced, as only 389 cases were recorded in 3 years. Additionally, 49.4% of dentists encountered a rare emergency case. This result is lower than the study of Alhamad *et al*. (2015) [2]. The previously mentioned authors accounted for 599 episodes of medical emergencies in 3 years, confronted by 67% of the respondents. In a study conducted in Brazil by Arsati *et al*. [5], 75% of dentists came across a medical emergency in the dental office within twelve months, higher than the medical emergency cases reported by the dentists in our study. We observed that approximately 72.7% of the dentists proclaimed the availability of emergency kits in their clinic, a higher value than in the study of Albelaihi *et al*. (2017) [1]. However, in their survey, Alhamad *et al*. (2015) [2] documented that most of the participants (74.3%) reported that they had emergency kits in their clinics. This study is corroborated by Hassan *et al*. (2018) [3] with 85% of respondents reporting having emergency drug kits in their clinics. Both these studies show higher values than our study findings.

Dealing with emergency cases was not an easy task among dental practitioners. Our study also found that only one-third of the dentists can handle emergencies, while 22.5% consult physicians [6–8]. Our results are in conjunction with the paper of Albelaihi *et al*. (2017) [1], where 37% of participants were confident to handle any medical emergency in the dental office. However, in a report by Alhamad *et al*. (2015) [2], the dentist showcased higher self-confidence in dealing with emergency cases by themselves (62.7%), and 26.2% would call a physician for assistance. On the other hand, another survey by Arsati *et al*. (2010) estimated that 24.3% of the dentists did not ask for help in a medical emergency, while 29% took assistance from some physician or another dentist (25.4%), slightly lower than our report.

It was observed that the most common medical emergency encountered by the dentist was vasovagal syncope (32.6%), followed by hypoglycemia (29.2%). Several papers reported vasovagal syncope and hypoglycemia as the two most typical medical emergencies in dental clinics [2, 9, 10]. A Brazilian study [5] differed in this regard, as they recorded orthostatic hypotension as the second most common medical emergency.

Incidentally, from the reports of this study, we learned that the most common available emergency drug at the dental clinic varies with each region. For instance, in our study, glucose source was the most frequently cited emergency drug (59.9%), followed by oxygen (56.6%) and adrenaline (42.7%). However, in the central region, Hassan and Qahtani [7] reported that aspirin was the most available emergency drug, followed by glucose and nitroglycerine. In the Western region, Albelaihi *et al*. reported [1] adrenaline as the conventional drug obtainable in emergencies, followed by antihistamines. While in the Eastern region (Alhamad *et al*., 2015) [2], oxygen, adrenaline, and glucose were the drugs readily available in the dental clinic for emergencies.

Dealing with a medical emergency requires a good amount of experience and additional courses in that particular field to increase knowledge. That is why it is vital to do hands-on courses for the cardiopulmonary resuscitation (CPR) program, which provides an auxiliary means to manage an ER case. In our study, 38.2% of the dentists rated attending CPR courses as very good, followed by fair (36.3%). This report did not seem to disagree with Alhamad *et al*.'s (2015) [2], as they documented that 40.7% of the dentists rated CPR courses very good, and 38% reported them as fair. On the other hand, dental students showed conflicting reports, as only 0.02% could perform CPR even though most showed some knowledge. They further concluded that aspiring Brazilian dentists are not fully prepared to manage medical ER and have insufficient experience in CPR [5], indicating that dental students need to gain more knowledge and confidence before performing CPR.

In our study, the self-competency in performing CPR was rated poor, as only 34.5% indicated that they are proficient in conducting CPR, similar to Alhamad *et al*. [2], who found only 45% to be efficient. We also noticed that dentists were not accustomed to using emergency drugs. 41.9% of the dentist had no confidence in using emergency drugs, which were congruent with the paper of Alhamad *et al*. (2015) [2], where one-third of respondents were either doubtful or did not know how to use ER drugs.

Some factors influenced the frequency of facing an emergency case, including gender (P=0.001), years of practice (P=0.007), and graduating in Saudi Arabia (P=0.012). Our study suggests that males who graduated from Saudi Arabia were significantly more likely to experience medical emergencies than those with fewer years of practice. However, according to Alhamad *et al*. (2015) [2], there were no statistically significant differences between males and females and between government and private dentists in the frequency of medical emergencies and other study variables.

The sampling methods and cross-sectional study design represented the drawbacks of this study. The number of participants in this study was small, and the results may not be representative for all dentists in the Riyadh region of Saudi Arabia. Furthermore, the patient sample size may not be enough to assess all Saudi Arabian hospitals. Future research is needed to achieve more solid findings. A web-based survey was also distributed to a select group of dentists whose email addresses had already been registered with the Saudi Dental Association.

Finally, even though the current study used Google search patterns to gauge the opinions of a broad group of dental professionals, some people do not utilize the Internet to look for available specialized information on medical emergencies.

## CONCLUSION

The research revealed that emergency kits were available in dental clinics but were not frequently needed. Furthermore, a considerable number of dentists were not fully experienced in handling the emergency. Self-competency in performing CPR was poor, and self-confidence in using ER drugs was also suboptimal. In this regard, a dentist should attend more courses on handling emergencies, performing CPR, and using emergency drugs in their dental practice. Further training on CPR is also necessary to increase their knowledge and perform better in emergencies. Dental institutions and educators of dental courses should develop a program to guide dental practitioners in dealing with emergencies.

## Supplementary Material



## Data Availability

Further data is available from the corresponding author on reasonable request.
