# Supplementary material for: Are dental professionals ready to deal with medical emergencies in their clinical office? A survey of university hospitals
Source: J Med Life. 2022 Aug;15(8):979–86. doi: 10.25122/jml-2022-0012 (PMC9514817; doi:10.25122/jml-2022-0012)
Supplement: Supplementary file 1 [file JMedLife-15-0979-s001.pdf]

Annex

Annex 1. Survey questionnaire.

**1.**	Do you have an emergency kit?
**2.**	How many emergency kits are there in the hospital?
**3.**	What is the content of the emergency kit?
**4.**	How many times periodical checking of the emergency kit is done?
**5.**	How many times do you use the emergency kit yearly?
**6.**	What is the most common drug used?
**7.**	What is the least common drug in use?
**8.**	How frequently do you replace the emergency kit?
**9.**	Has an emergency case occurred in your clinic?
**10.**	How many emergency cases occurred in your clinic?
**11.**	How many times have you use the emergency kit?
**12.**	Level of knowledge about the emergency kit and its contents?
**13.**	Did you take any courses regarding emergency medicine?
